# Transmembrane protein rotaxanes reveal kinetic traps in the refolding of translocated substrates

**DOI:** 10.1038/s42003-020-0840-5

**Published:** 2020-04-03

**Authors:** Jianfei Feng, Pablo Martin-Baniandres, Michael J. Booth, Gianluca Veggiani, Mark Howarth, Hagan Bayley, David Rodriguez-Larrea

**Affiliations:** 1 Department of Chemistry, University of Oxford, 12 Mansfield Road, Oxford, OX1 3TA UK; 2 Department of Biochemistry, University of Oxford, South Parks Road, Oxford, OX1 3QU UK; 3 Department of Biochemistry and Molecular Biology (UPV/EHU), Biofisika Institute (CSIC, UPV/EHU), Barrio Sarriena s/n, 48940 Leioa, Spain

**Keywords:** Single-molecule biophysics, Kinetics

## Abstract

Understanding protein folding under conditions similar to those found in vivo remains challenging. Folding occurs mainly vectorially as a polypeptide emerges from the ribosome or from a membrane translocon. Protein folding during membrane translocation is particularly difficult to study. Here, we describe a single-molecule method to characterize the folded state of individual proteins after membrane translocation, by monitoring the ionic current passing through the pore. We tag both N and C termini of a model protein, thioredoxin, with biotinylated oligonucleotides. Under an electric potential, one of the oligonucleotides is pulled through a α-hemolysin nanopore driving the unfolding and translocation of the protein. We trap the protein in the nanopore as a rotaxane-like complex using streptavidin stoppers. The protein is subjected to cycles of unfolding-translocation-refolding switching the voltage polarity. We find that the refolding pathway after translocation is slower than in bulk solution due to the existence of kinetic traps.

## Abstract (web summary)

Feng et al address the technical challenge of monitoring the protein folding during membrane translocation. Using thioredoxin as a model and with electric potential driving its translocation through single a-hemolysin nanopore, they observe that after translocation, folding is slower than in bulk due to existence of kinetic traps.

## Introduction

Protein folding has attracted much attention during the last 50 years, because it is central to cell function and disease. Early studies examined how proteins folded in vitro, typically after chemical or thermal denaturation^1,2^. More recently, technical developments have allowed a deeper understanding of protein folding under experimental conditions closer to those found in vivo^3–7^. A relevant event within the cell is the emergence of a polypeptide from the ribosome exit tunnel^5,8^. The nascent chain emerges N terminus-first and, before the C terminus is complete, the protein may start folding^9,10^, which is a strikingly different process to experimental refolding in bulk solution^4^. Second, during or after synthesis by ribosomes, proteins may be transported between compartments in the cell^11^ or into the extracellular medium^12^. During these processes, they must cross membranes by using one of a diverse set of translocons. For example, in bacteria alone, there are at least ten secretion systems^13,14^ and translocated substrates include many of the virulence factors secreted by pathogens^14^. Usually, secretion systems translocate unfolded polypeptides, and therefore the substrate must refold after or during the translocation process^15–17^.

Currently, pulse-chase experiments, which have a temporal resolution of minutes, are often used to study the folded state of proteins following membrane translocation in cells^18,19^. Similar temporal resolution is available when experiments are performed in vitro with reconstituted translocon components^20^. Further, these ensemble experiments frequently fail to resolve the elementary steps of complex processes^21^. Studies of translocation to the periplasm of substrates that contain disulfides have been a remarkable exception^12^. By using a mutant of DsbA, a disulfide-bond-forming enzyme, the formation of covalent mixed-disulfide complexes between DsbA and a polypeptide substrate was slowed, which allowed the resolution of intermediates in the folding process. However, this approach is limited to disulfide-containing substrates in bacteria.

We recently introduced a method to study co-translocational protein unfolding at the single-molecule level^22–25^, which avoids the requirement to synchronize an ensemble measurement. To achieve this, a single α-hemolysin pore (αHL) is inserted into a lipid bilayer. The αHL pore forms a conduit of ~2 nm diameter (~1.3 nm at its narrowest point), which can accommodate alpha helices but forces a protein to pass unfolded^26^. A model protein, thioredoxin (Trx), derivatized at one of its termini with a negatively-charged oligonucleotide, is driven into the pore by an electric field, which causes unfolding and translocation of the protein^22,23^. Steps in the ionic current carried by the pore represent intermediates in the translocation process^27^. In an alternative approach, a signal sequence is placed at the C terminus of a polypeptide and recognized by the molecular motor ClpX, which drives translocation^24,25^. Both approaches allow real-time tracking of the unfolding and translocation process. However, following translocation, the protein substrate is released into the *trans* compartment and cannot be further examined to determine whether it has refolded.

Here, we generate a rotaxane-like structure that prevents release of the protein substrate into the medium^28^ and allows examination of the folding status of the translocated substrate. The overall process involves unfolding, translocation and refolding of the protein substrate. Devoid of molecular motors and chaperones, our model system constitutes a simplification of the more complex in vivo process but has the vectorial nature of protein folding as in vivo. We first attached 3′-biotinylated oligonucleotides to each terminus of Trx. The oligonucleotide-Trx-oligonucleotide conjugate with one end bound to monovalent streptavidin (mSA)^29^ is driven into the nanopore by an applied potential. The direction of translocation (N-to-C or C-to-N) is ascertained by using distinguishable oligonucleotides. The oligonucleotide that reaches the *trans* side of the bilayer is captured by a second streptavidin (SA). The interlocked architecture of the threaded complex bound to one streptavidin at each side of the membrane constitutes a transmembrane protein rotaxane and is stable until either streptavidin dissociates. The protein substrate can then be driven from one side of the membrane to the other by repeatedly switching the polarity of the voltage between positive and negative. Our approach allows an examination of the evolution of the folding landscape of Trx, beginning within milliseconds after translocation.

## Results

### Identification of the terminus leading protein translocation

**Fig. 1:**
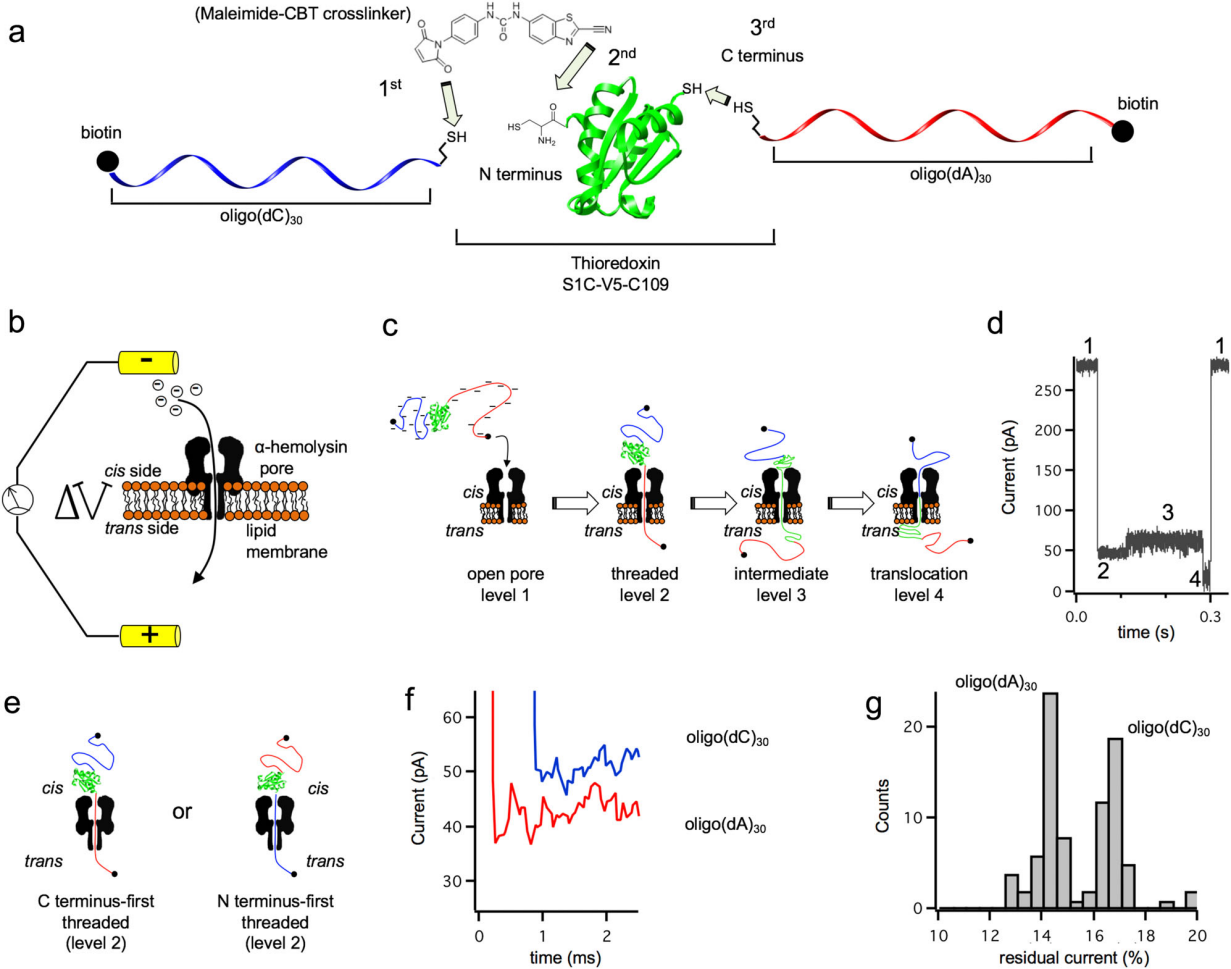
Differential tagging and co-translocational unfolding of Trx V5. **a** Synthesis of oligo(dC)_30_-V5-oligo(dA)_30_. After each of the three steps, the product was purified and characterized by SDS-PAGE and MS (Supplementary Figs. 1–3). **b** An electric potential across a lipid bilayer containing a single αHL pore (*cis* at ground). In response to an applied positive potential, anions and cations are driven through the pore, which is recorded as a positive current. **c** When oligo(dC)_30_-V5-oligo(dA)_30_ is added to the *cis* compartment at +140 mV, one of the negatively-charged oligonucleotides is pulled into the αHL pore (level 2). The electrophoretic force acting on the oligonucleotide partly unfolds the protein (level 2→level 3). A relatively long-lived intermediate is generated in which the terminus of the polypeptide chain threaded through the pore (level 3). At this point, the remaining folded portion of Trx is unstable and, after a waiting time (level 3→level 4), it spontaneously unfolds and diffuses through the pore (level 4). **d** Electrical recording of a single translocation event showing levels 1–4. **e** Oligo(dC)_30_-V5-oligo(dA)_30_ can thread either N or C terminus-first. **f** Ionic current signals of level 2 for entry with the N terminus-first (blue) and C terminus-first (red). **g** Event histogram of the residual currents (*I*_res%_) in level 2 during multiple translocation events of oligo(dC)_30_-V5-oligo(dA)_30_ through a single αHL pore showing two populations. The *I*_res%_ in level 2 was used to define the direction of translocation. The result was confirmed with two additional pores.

We used a mutant of *E. coli* thioredoxin 2 (PDB code 2TRX) lacking the natural disulfide bond (C32S-C35S), with three mutations that increase stability (A22P-I23V-P68A)^30^ and both an N-terminal and a C-terminal cysteine (S1C and the added C109). This mutant, V5, was selectively modified on the N-terminal cysteine by using a click reaction with a 2-cyanobenzothiazole (CBT)^31^ to form a stable thiazoline ring^32^. To introduce the CBT functionality into a thiol-modified oligonucleotide, we coupled the amino group of 6-amino-2-CBT to *p*-maleimidophenyl isocyanate (Supplementary Fig. 1a, b). The product, a maleimide-CBT crosslinker, was reacted with the 5′-thiol of a 3′-biotinylated oligonucleotide composed of 30 cytosines, and the resultant 5′-CBT-oligo(dC)_30_-biotin-3′ was purified by ion-exchange chromatography (Fig. 1a and Supplementary Fig. 1c–f).

Following bacterial expression and purification of S1C-V5-C109, more than half of the N-terminal cysteines were found modified with pyruvate^33^, which was removed by incubation with methoxyamine exposing the 1,2-aminothiol (Supplementary Fig. 2). Subsequent incubation of the protein, reduced with TCEP, with 5′-CBT-oligo(dC)_30_-biotin-3′, followed by ion-exchange chromatography, produced a product that gave a single band of ~20 kDa upon electrophoresis (Supplementary Fig. 3a, d, f), consistent with a single oligonucleotide coupled to the N terminus of S1C-V5-C109^31^. Because CBT reacts site-specifically with 1,2-aminothiols, the C-terminal cysteine was not altered. The conjugate was then reacted at the C-terminal cysteine with the 5′-thiol of a 3′-biotinylated oligonucleotide, composed of 30 adenines, that had been activated with 2,2′-dipyridyl disulfide (Supplementary Fig. 3b, c)^34^. The purified product produced a band of ~30 kDa upon electrophoresis (Supplementary Fig. 3f), and the native mass spectrometry (native MS) was consistent with S1C-V5-C109 derivatized with different oligonucleotides at the N and C termini (hereafter V5 stands for the S1C-A22P-I23V-P68A-C32S-C35S-C109 mutant, and oligo(dC)_30_-V5-oligo(dA)_30_ for the mutant modified with the oligonucleotides, Supplementary Fig. 3g). One of the amide bonds of the maleimide group was hydrolyzed to completion during the synthesis of oligo(dC)_30_-V5-oligo(dA)_30_, which produced a + 18 peak in the MS but left the second amide and the linkage intact.

We added oligo(dC)_30_-V5-oligo(dA)_30_ to the *cis* side of a phospholipid bilayer containing a single αHL pore. Under an applied potential of +70 to +140 mV, the construct was driven into the pore, by either of the two oligonucleotides, causing the unfolding and translocation of the protein. Previously we have shown that an oligonucleotide tag at the C-terminus does not modify the stability of the native state as judged by the similar urea induced unfolding curve, but it only serves to drive the translocation^22^. The ionic current passing through the pore revealed the various molecular steps of the process^23^ (Fig. 1b–d). First the leading oligonucleotide, oligo(dC)_30_ for N terminus-first or oligo(dA)_30_ for C terminus-first, threaded into the pore (level 2 in Fig. 1e). The ionic current differed depending on the composition of the oligonucleotide, with oligo(dC)_30_ giving a higher residual conductance value than oligo(dA)_30_ (*I*_res%_ = 16.5 ± 0.5% (*n* = 36) and *I*_res%_ = 14.2 ± 0.2% (*n* = 43) at +140 mV, respectively, Fig. 1f, g), in accord with previous studies with immobilized oligonucleotides^35,36^. The ionic current during level 2 can therefore be used to determine the orientation in which oligo(dC)_30_-V5-oligo(dA)_30_ threads through the pore for each event that is registered. After threading, the protein unfolded by way of a long-lived intermediate (level 3 in Fig. 1c, d) that spontaneously unfolded and then passed through the pore by diffusion^22^ (level 4 in Fig. 1c, d). Once the leading oligonucleotide has entered the *trans* compartment and left the electric field, there is no longer a pulling force on the protein. Following the translocation of the remaining unfolded protein, the pore remained open until another oligo(dC)_30_-V5-oligo(dA)_30_ entered. The terminal oligonucleotide translocated too rapidly to generate a distinguishable ionic current level. The dwell time and ionic current of level 3 also depended on the direction of translocation, being longer and noisier if co-translocational unfolding is initiated through the N terminus, as already reported^23^. These features can also be used to determine the direction in which oligo(dC)_30_-V5-oligo(dA)_30_ initiates translocation, and the directionality of pore entry based on the *I*_res%_ values in level 2 are in agreement with the features observed in level 3. We study the C-first translocation reaction because when the protein is pulled back the protein translocates N-terminus first and therefore vectorial refolding is as in most biological systems. For these molecules, the dwell time in level 2 reflects the unfolding kinetics of a C-terminal region^22,23^, and the dwell time in level 3 reflects the unfolding kinetics of the partly unfolded intermediate (Fig. 1c, d). The rate constants determined here (k_2→3_ = 6.9 ± 0.5 s^−1^ and k_3→4_ = 1.0 ± 0.1 s^−1^ at +100 mV, *n* = 160, Supplementary Fig. 8a, b) are in good agreement with those reported^22^ for the construct C1S-V5-C109-oligo(dC)_30_ (k_2→3_ = 6.0 ± 1.0 s^−1^ and k_3→4_ = 1.5 ± 0.1 s^−1^ at +100 mV). These results suggest that simultaneous N-terminal and C-terminal modification have a minimal effect on the kinetics of co-translocational protein unfolding.

### Formation of a transmembrane protein rotaxane

**Fig. 2:**
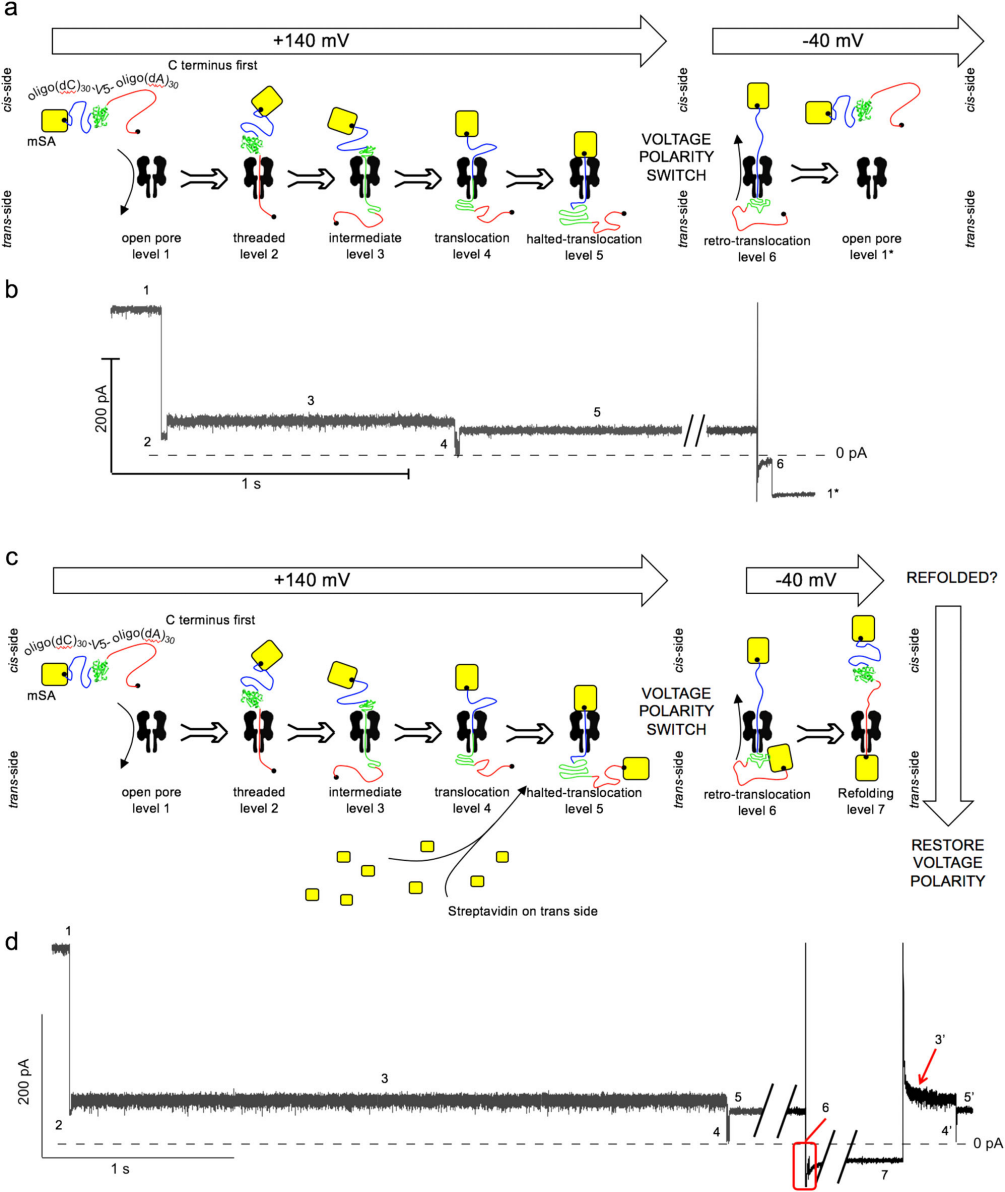
Formation of an oligo(dC)_30_-V5-oligo(dA)_30_/αHL rotaxane. **a** Prevention of release into the *trans* compartment. Oligo(dC)_30_-V5-oligo(dA)_30_ (biotinylated oligonucleotides) was treated with a sub-stoichiometric amount of mSA. A complex with one of the two oligonucleotides bound to mSA threads through the pore at +140 mV with the C terminus free oligonucleotide first. After unfolding and translocation of the protein (Trx V5), the translocated substrate remains arrested in the pore by the mSA. Reversal of the voltage polarity (−40 mV) drives the complex back into the *cis* compartment (retro-translocation), where it is released to the solution. The pore is left at level 1* (the open state at a negative potential). **b** Ionic current signal corresponding to the events shown in ‘a’. Level 1, open αHL pore; level 2, oligonucleotide threaded into the pore; level 3, unfolding intermediate; level 4, translocation (largely diffusive) of the unfolded protein; level 5, second oligonucleotide threaded into the pore; level 6, retro-translocation; level 1*, open pore (−40 mV). **c** Formation of a transmembrane protein rotaxane. The steps described in ‘a’ were carried out with SA to the *trans* compartment. After the protein translocation, an approximately 5 min delay allowed SA to bind to the biotinylated oligonucleotide. Then, after a voltage step to −40 mV, the protein was retro-translocated but remained associated with the αHL pore as a rotaxane. After a delay, a subsequent step to +140 mV can be used to probe the folded state of the Trx V5 in the *cis* compartment. **d** Ionic current signal characteristic of rotaxane formation. Level 1, open αHL pore (+140 mV); level 2, oligonucleotide threaded into the pore; level 3, unfolding intermediate; level 4, translocation of the unfolded protein; level 5, second oligonucleotide threaded into the pore; level 6, retro-translocation; level 7, initial leading oligonucleotide threaded into the pore. During level 7 (here 0.49 s) the protein refolds in the *cis* compartment and when the potential is stepped back to +140 mV, levels 2′, 3′, 4′, and 5′ appear. The prime symbol signifies that the levels are generated not from the native Trx, but from a refolded form.

Oligo(dC)_30_-V5-oligo(dA)_30_ present in the *cis* compartment was then treated with a sub-stoichiometric amount of mSA. Each of the two oligonucleotides carried a terminal biotin and, therefore, a significant fraction of the construct bound only a single mSA^29^, leaving the other terminus free. When these complexes were pulled into the pore by voltage (+100 to +140 mV), they produced the characteristic ionic current signature that represents threading, unfolding and translocation of the protein (Fig. 2a, b). However, following translocation, the ionic current signal does not recover to the open pore level but remains at a new level (level 5, in Fig. 2a, b). The residual current in level 5 also depended on whether the process was initiated N or C terminus-first, with I_res%_ = 17.8 ± 0.1% (*n* = 33, +140 mV) and *I*_res%_ = 17.4 ± 0.1% (*n* = 32, +140 mV), respectively (Supplementary Fig. 4). During level 5, mSA on the *cis* side atop the pore stops the movement of the construct, with the terminal oligonucleotide threaded into the pore^35^. The immobilized oligonucleotides were in the 5′-end-first orientation in level 5, and the higher *I*_res%_ values for the oligo(dA)_30_ blockades than for those arising from the oligo(dC)_30_ blockades, opposite to the result in level 2 (3′-end-first), is in agreement with previous work^35,36^.

The protein was hence in the *trans* compartment, and when the potential was changed from +140 mV to −40 mV the complex was pulled in the opposite direction, from *trans* to *cis*, i.e., it was forced to retro-translocate. At the beginning of this voltage step, the ionic current signal moved transiently to a new level 6, before the characteristic ionic current level of the open pore at −40 mV was obtained (level 1* in Fig. 2a, b, the asterisk signifies the negative potential). Level 6 therefore represents the unfolding and translocation in the *trans* to *cis* direction.

Alternatively, after oligo(dC)_30_-V5-oligo(dA)_30_ threaded C terminus-first, instead of stepping the voltage to −40 mV we added mSA to the *trans* compartment, which bound to the biotinylated oligo(dA)_30_ that had led the translocation process (Fig. 2c). Hence, a rotaxane-like structure was generated (level 5 in Fig. 2c, d)^28^. In this state, a voltage step from +140 to −40 mV did not produce the current level of the open pore (level 1*), but instead the signal assumed a new level 7 in which the translocating Trx V5 is presumed to have moved completely back into the *cis* compartment, and the C-terminal oligonucleotide is threaded through the pore. After a selected time in level 7, the potential was switched back to +140 mV, and we observed an ionic current pattern similar to that produced during the initial translocation, with levels 2′, 3′, 4′, and 5′ (Fig. 2d). Importantly, the time provided in level 7 was time available to allow the protein to refold, and the dwell times during subsequent forward re-translocation (levels 2′ and 3′) revealed the kinetic stability of the refolded state. Finally, and in agreement with the proposed model, when the voltage was switched from +80 mV (a lower voltage to produce an extended level 2′) to −40 mV during residence in level 2′ or 3′ (i.e., while the protein was still or largely still in the *cis* compartment), level 6′ was not observed, but rather level 7 reappeared (Supplementary Fig. 5a, c). Similarly, if the voltage was switched from −40 mV to +80 mV during level 6′ (i.e., while the protein was largely in the *trans* compartment), levels 2′, 3′ and 4′ were never observed (Supplementary Fig. 5a, d).

These results are consistent with the formation of a transmembrane protein rotaxane. In this state, the protein can be translocated forwards and backwards across the pore by changing the polarity of the applied potential (Fig. 2c) and thereby subjected to multiple cycles of unfolding, translocation and refolding, observable with millisecond time resolution. Yet higher temporal resolution is challenging, because of capacitive transients after the voltage steps. In addition, the use of monovalent traptavidin (mTA)^37^ in the *cis* compartment, which dissociates from biotin more than ten-times slower than mSA, and traptavidin (TA) in the *trans* compartment, allowed over 24 h of recording on the same individual rotaxane.

### The refolding landscape of a translocated protein substrate

**Fig. 3:**
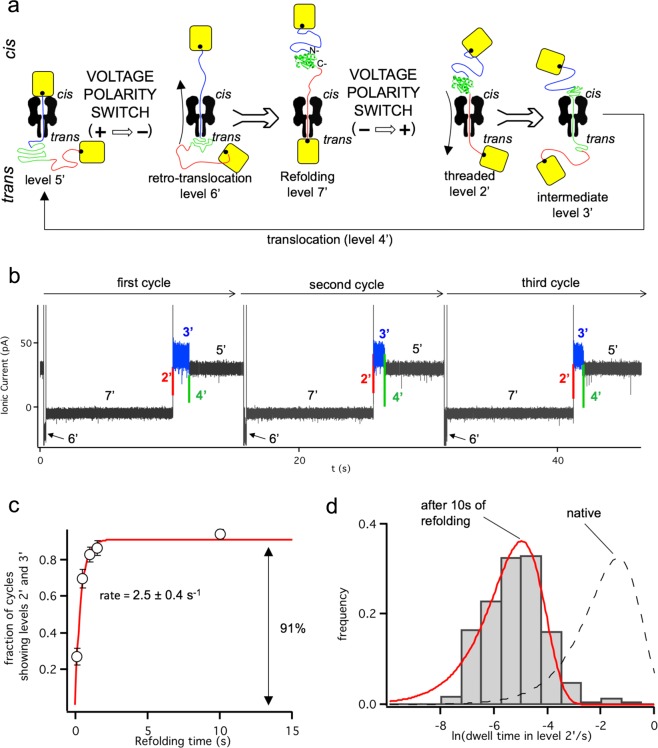
Employing transmembrane protein rotaxanes to analyze protein refolding. **a** Experimental configuration. Once the rotaxane has been formed (level 5′), the protein is moved from the *trans* to the *cis* side by applying a negative potential (level 6′→level 7′). The retro-translocated substrate remains in level 7′ for a specified refolding time, which is computer controlled. Subsequently, the potential is switched to +100 mV, and the co-translocational unfolding of the refolded protein is recorded (level 2′→3′→4′→5′). This process is then repeated. **b** Ionic current signal from three cycles as described in ‘**a**’ with a refolding time of 10 s. **c** The fraction of cycles in which the refolded substrate shows both levels 2′ and 3′, characteristic of refolding to a native-like state, increases with refolding time. The increase of the folded fraction fits a single exponential (red) yielding a rate constant of 2.5 ± 0.4 s^−1^. Each point represents data from ~300 translocation events acquired with three different pores. **d** Frequency histogram of the dwell times in level 2′ observed during 345 cycles with a refolding time of 10 s (data collected with three different pores). The data are well described by an exponentially-distributed population of dwell times (red solid line, k_2’→3’_ = 145 ± 10 s^−1^; black dashed line: probability density function for the native state, k_2→3_ = 6.9 ± 0.5 s^−1^).

We next aimed to define the folding landscape of Trx after translocation through the αHL pore, by subjecting the protein in the rotaxane to cycles of unfolding, translocation and refolding (Fig. 3a, b). In this work, we analyze constructs that enter C terminus-first from the *cis* compartment. Therefore, the refolding we investigate is initiated during or after retro-translocation N terminus-first from the *trans* into the *cis* compartment.

The protein may refold outside the pore during level 7′ (Fig. 3a). We held the protein at level 7′ for a selected period (the refolding time) and then switched to a positive potential (+100 mV) to probe the state of the protein^38^, which at this point could have returned to the native state, remained in an unfolded state or be in an intermediate state. By comparing the dwell times in levels 2′ and 3′ to the corresponding dwell times during the initial unfolding of the native protein (levels 2 and 3), we analyzed the stability of individual refolded Trx molecules. The dwell time in level 4′, which represents diffusive translocation of the remaining unfolded polypeptide, was unaffected by the refolding time and was similar to that of level 4 of the native protein (Supplementary Fig. 6). We allowed the protein to refold at level 7′ for 0.09 s, 0.49 s, 0.99 s, 1.49 s, 10 s, 100 s, or 1000 s, and probed the state of individual Trx molecules hundreds of times under each condition.

When we allowed the translocated Trx V5 protein molecule to refold for 90 ms, levels 2′ and 3′ were observed in only 99 out of 365 re-translocation events (Fig. 3c). In the remaining cases, level 2′ (which has been previously related to the folding of ~40 aminoacids at the C terminus^22,23^), level 3′ (previously related to the fold of the remainder of the polypeptide chain^22,23^) or both were absent (Supplementary Fig. 7). The absence of these levels either represents a lack of folding or the formation of weakly folded regions that unfold faster than the detection limit (~0.2 ms). Importantly, we observed that the longer the refolding time, the more frequently the signal contained both levels 2′ and 3′ (Fig. 3c). The time course of this folding process (measured as the presence of both levels 2′ and 3′) was not limited by the translocation of the unfolded polypeptide (that is ~50 fold slower than the folding event, Supplementary Fig. 6) and well described by a single exponential function, yielding a rate of 2.5 ± 0.4 s^−1^.

After 10 s of refolding, a plateau was reached where ~90% of the re-translocation events showed both levels 2′ and 3′. Nonetheless, the co-translocational unfolding of the refolded Trx V5 was faster than that of the Trx V5 that was unfolded for the first time (Fig. 3d). We observed a shorter dwell time in level 2′, which converted to level 3′ with a rate constant of k_2’→3’_ = 250 ± 10 s^−1^ (at + 100 mV, *n* = 345), compared to the value for the native Trx V5 of k_2→3_ = 6.9 ± 0.5 s^−1^ (at + 100 mV, *n* = 160). The dwell time in level 3′ was also shorter, but the difference was less obvious (less than 7-fold reduction, Supplementary Fig. 8).

These results imply that Trx V5 had not reached the native state after 10 s of refolding. The folding kinetics of Trx are complicated because there is an invariant *cis* proline, Pro-76, buried in the native structure. In bulk solution, when Trx is placed under denaturing conditions for a few seconds, proline residues do not have time to isomerize and refolding is fast (sub-millisecond)^39,40^. When Trx is left under denaturing conditions for hundreds of seconds, proline isomerization occurs and Trx then refolds slowly (*t*_1/2_ ~ 500 s)^41^. In this respect, the unfolding and translocation of Trx V5 through the pore typically takes less than ~1.5 s. This gives little time for proline isomerization to occur (proline isomerization takes from 10^3^ s -*cis* to *trans*- to 10^4^ s -*trans* to *cis*-^42^, roughly once every 1000 cycles), and we therefore expected refolding to occur in the sub-millisecond regime. Given that 10 s after translocation the the refolded Trx V5 remains in a state which unfolds faster than the native Trx V5, we conclude that the folding mechanism differs from the fast folding route that occurs in bulk solution.

We next used oligo(dC)_30_-V5-oligo(dA)_30_ to explore the folded state of Trx V5 after prolonged folding times. We first confirmed that Trx V5 mutant is a slow folder in bulk solution by measuring the recovery of tryptophan fluorescence after dilution from 4 M guanidinium chloride. Refolding proceeded at 2.3 × 10^−3^ s^−1^, a similar rate to that of wild-type Trx^30,39–41^, showing that in these cases folding is rate-limited by proline isomerization (we could not use the tagged substrate because we do not generate enough sample). By contrast, after retro-translocation through the nanopore and a 1000 s delay for refolding, we only observed ~15% of the molecules unfolding with dwell times in levels 2′ and 3′ that were characteristic of the native state (Supplementary Fig. 8). This yields a refolding rate of 1.4 × 10^−4^ s^−1^, >15-fold slower than that observed in bulk solution, which unlike refolding upon translocation requires proline isomerization. In atomic force microscopy and optical tweezer experiments, tethering to a surface, a cantilever or a bead slows down folding due to dragging forces^43^. We expect this effect to be negligible in our case: one terminus of the protein is anchored to the pore embedded in the lipid bilayer and the other is tethered to an oligonucleotide-streptavidin complex, neither of which produces a strong drag. On the other hand, the N-terminal and C-terminal of Trx are fully exposed to the solvent, suggesting that the addition of the tags should have a minor effect on the kinetics. Therefore, refolding after protein translocation conforms with neither the fast folding nor the slow folding pathways observed for Trx in bulk solution.

**Fig. 4:**
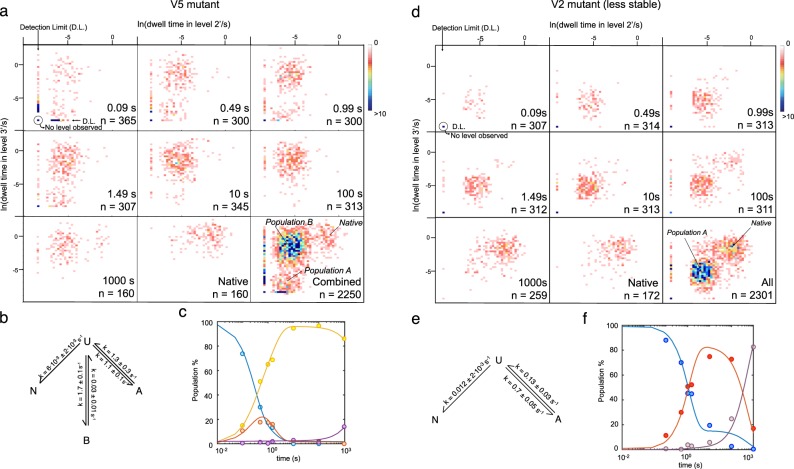
Folding landscapes of translocated protein substrates. **a** Bivariate histograms representing the dwell times in levels 2′ and 3′ for oligo(dC)_30_-V5-oligo(dA)_30_ threaded C terminus-first. Data were acquired with three different pores. Each panel corresponds to the indicated refolding time (n: number of events). When level 2′ or 3′ was not observed, a dwell time of 0.2 ms, the temporal detection limit, was assigned. The “Native” panel records values obtained from Trx V5 that had never been unfolded. The “All” panel displays the data from all the other panels in a single plot to provide clearer visualization of three populations. **b** Kinetic model giving the lowest AIC and BIC values with the kinetic constants derived from the fit. **c** Time-course evolution of population A (red circles), population B (yellow circles), native Trx V5 (purple circles) and unfolded Trx V5 (blue circles). The solid lines represent the best fits to the model with the lowest AIC and BIC values. **d** Three destabilizing mutations were introduced into Trx V5 to form Trx V2. A rotaxane was formed, and the refolding was examined as in ‘**a**’. When level 2′ or 3′ was not observed, a dwell time of 0.2 ms was assigned. **e** Kinetic model giving the lowest AIC and BIC values with the kinetic constants derived from the fit. **f** Time-course evolution of population A (red circles), native Trx V2 (purple circles) and unfolded Trx V2 (blue circles). The solid lines represent the best fits.

An advantage of our approach is that a given refolded state can be characterized with at least as many variables as the number of observable unfolding steps, which allows the use of bivariate scatter plots to improve the ability to distinguish different populations and better resolve the folding landscape. We display dwell times in levels 2′ and 3′ for the refolding times of 0.09 s, 0.49 s, 0.99 s, 1.49 s, 10 s, 100 s, and 1000 s acquired from three different rotaxanes and the superimposed data to better visualize the identified populations (Fig. 4a). The plots are snapshots of how the protein moves through the folding landscape. Trx V5 emerged unfolded from the pore, and after 0.09 s, ~73% of the events examined lacked any detectable folding (level 2′ or 3′). The remainder (~27%) had entered one of two states, population A or population B, both less stable than the native state based on the shorter dwell times in levels 2′ and 3′. During the first second (0.99 s), the fraction of events with undetectable folding was reduced drastically to ~17%, while population A remained constant (from ~10% at *t* = 0.09 s to ~17% at *t* = 0.99 s) and population B increased (from ~15% at *t* = 0.09 s to ~65% at *t* = 0.99 s). As the population with undetectable folding disappeared, so did population A. After 10 s of refolding, most of the events were found within population B, and the native state became slowly populated, reaching ~15% at 1000 s.

To set our analysis on a more quantitative basis, we used *k*-means clustering^44^ to classify the events into population A, population B, or the native state (Supplementary Fig. 9). When either level 2′, level 3′ or both were absent, the events were assigned to an unfolded state. By using this approach, we found that the native state was characterized by k_2’→3’_ = 6.2 s^−1^ and k_3’→4’_ = 1.1 s^−1^ (at + 100 mV, *n* = 2251), which are close to the values obtained by fitting 1D histograms to exponential distributions (k_2→3_ = 6.9 s^−1^ and k_3→4_ = 1.0 s^−1^ at +100 mV, *n* = 160 respectively, Supplementary Fig. 8a, b). Next, the ability of various kinetic models describing the time-dependent evolution of the populations obtained by *k*-means clustering was evaluated based on the Akaike information criterion (AIC)^45^ and the Bayesian information criterion (BIC)^46^ (Supplementary Fig. 10). These are estimators that allow model selection based on information theory. Remarkably, the best-fitting model was the one that imposed an equilibrium between the unfolded state and both populations A and B (Fig. 4b, c), which suggests that both populations A and B are kinetic traps and that successful folding only occurs from the unfolded state. While we do not know the exact structural nature of these intermediates, the folding funnel hypothesis predicts that along the folding pathway the protein would acquire more native-like structure as the free energy decreases^47^. We therefore take the recovery of mechanical stability as a proxy of nativeness. Similarly, folding upon emergence from the ribosome exit tunnel during protein synthesis may lead to folded but non-native structures^5^. A similar result was reported for the α-lytic protease, which in the absence of its pro-region folds to a native-like structure with its three disulfides in place that lacks the activity and stability of the native state^48^.

To seek support for this interpretation, we performed the same experiments on a Trx mutant of lower thermodynamic stability. We assume that the native state is the deepest well in the free energy landscape. We also assume that intermediates share structural similarities with the native state. A final assumption is that transition states between one state and another share structural features of them both. Based on these assumptions, it follows that destabilization of the native state should cause slower folding kinetics in the presence of on-pathway intermediates^49,50^. Conversely, destabilization would result in faster folding kinetics if the observed intermediates are off-pathway. We removed the stabilizing mutations A22P-I23V-P68A from Trx V5, which increase the unfolding temperature by 10 °C^30^ to give Trx V2, and then formed a transmembrane protein rotaxane that was subjected to cycles of unfolding, translocation and refolding. This less stable mutant behaved similarly to Trx V5 but co-translocational unfolding of the native state was faster (k_2→3_ = 11.6 ± 0.4 s^−1^ and k_3→4_ = 7.5 ± 0.3 s^−1^ at +100 mV, *n* = 172), as expected^22^. Refolding, in agreement with the kinetic trap interpretation, was also faster. After 1000 s, Trx V2 had recovered native state stability in ~83% of the cycles examined. The refolding rate was 1.8 × 10^−3^ s^−1^, whereas that of Trx V5 was 1.4 × 10^−4^ s^−1^. The kinetic model that best fitted the data for Trx V2 again requires that the observed intermediate is off-pathway (Fig. 4d–f and Supplementary Figs. 11, 12). Therefore, in the nanopore system explored here, upon vectorial emergence from the pore, Trx rapidly collapses to non-native state(s) that shows moderate stability and is off-pathway. Due to the presence of both levels 2′ and 3′, these states could have similar folds to the native state, nevertheless they must return to the unfolded state to be converted to the native state.

## Conclusion

We have presented a single-molecule approach that, through the formation of a protein rotaxane in a transmembrane pore, allows the evaluation of the folded state of a protein at different refolding times after membrane translocation. The approach has millisecond temporal resolution and allows for characterization of the pathway to the refolded state with more than one parameter, which provides a better resolved folding landscape. Another important facet of our study, by comparison with recent single-molecule methods that examine the folding landscape of proteins^51,52^, is that the protein emerges vectorially from the pore as it does from a ribosome or translocon although in our case the rate may be 2–3 orders of magnitude faster. The approach requires repeated oligonucleotide-driven translocation and retro-translocation of the protein, which cannot be achieved by using a motor protein^24,25^.

We find that the folding mechanism of Trx following N terminus-first membrane translocation differs from that observed in bulk solution upon the removal of a denaturant such as urea or guanidinium chloride. In the latter, folding proceeds through a short-lived molten globule intermediate, if the protein has been in the unfolded state for only a few seconds^40^. If the protein is unfolded for hundreds of seconds, allowing proline isomerization to occur, folding is far slower and multiple intermediates are observed, all of them on pathways to the native state^41^. By contrast, in our nanopore approach, the protein is in the unfolded state only during the period of retro-translocation (tens of milliseconds), which implies that proline isomerization does not occur and hence is unlikely involved in the subsequent refolding process. We therefore would expect the rapid folding behavior: if the protein does not fold vectorially during retrotranslocation, once it completes translocation it should rapidly equilibrate in the unfolded state^53^ and the rate of folding should approximate that of the fast folding pathway observed in bulk solution. Given that we observed slow folding, the folding mechanism must be different from that in bulk solution, because the refolding rates are more than an order of magnitude slower. We show that this is a consequence of detours to off-pathway intermediates. We therefore suggest that the folding landscape we are observing is caused by the vectorial folding of the polypeptide as it emerges from the pore.

Our results are in line with analyses of T4 lysozyme^4^, HemK^5^, and flavodoxin^54^ which were artificially stalled as they emerged from the ribosome exit tunnel. For these particular cases at least, folding while tethered to the ribosome from the c-terminus is slower than in bulk solution. This suggests that the vectorial nature of folding inherent to protein synthesis and membrane translocation may increase the probability of folding intermediates including off-pathway ones. The generality of this phenomena and how chaperones modulate vectorial folding remain interesting yet unaddressed questions.

Finally, the methodology presented here can find interesting uses not only to study membrane associated processes at the single-molecule level but also in biotechnology applications, providing a novel way to prepare arrays of nanopore sensors.

## Methods

### Synthesis of maleimide-CBT crosslinker

17.5 mg of 6-amino-2-cyanobenzothiazole (CBT, Sigma) (0.1 mmol) and 10.7 mg of *p*-maleimidophenyl isocyanate (Sigma) (0.05 mmol) were dissolved in 1 mL of anhydrous dimethyl sulfoxide and stirred for 30 min at room temperature. The product was purified by preparative high-performance liquid chromatography (HPLC) on an Agilent 1260 Infinity system equipped with a Supelcosil PLC-18 12 μm, 250 × 21.2 mm column. The mobile phase consisted of 0.1% formic acid in H_2_O (buffer A), and 0.1% formic acid in acetonitrile (buffer B). A gradient from 5 to 95% of buffer B in buffer A was run over 30 min at a flow rate of 5 mL min^−1^. The separation was monitored at 335 nm. The identity of the product was confirmed by mass spectrometry (MS) and nuclear magnetic resonance (NMR) (Supplementary Fig. 1a, b).

### Oligonucleotide modifications

1.0 mg of an oligonucleotide (Biomers) composed of 30 cytosines with a 5′-thiol (hexamethylene linker) and a 3′-biotin (triethylene glycol spacer) was dissolved in 1 mL of 10 mM Tris·HCl, 1 mM EDTA, 5 mM TCEP, pH 7.5. After heating at 65 °C for 1.5 h, TCEP was removed with an Amicon Ultra column (0.5 mL, 3 kDa). The reduced oligonucleotide was then treated with the maleimide-CBT crosslinker (15 equivalents dissolved in a minimal volume of dimethyl sulfoxide) at room temperature for 2 h. The modified oligonucleotide (5′-CBT-oligo(dC)_30_-biotin-3′) was purified by ion-exchange chromatography on a monoQ FF Sepharose column (GE Healthcare) with an AKTApurifier FPLC (GE Healthcare) eluted with 0–1 M KCl in TE buffer (10 mM Tris·HCl, 1 mM EDTA, pH 8.0). The identity of the product was confirmed by MS (Supplementary Fig. 1c–f).

5′-S-thiopyridyl-oligo(dA)_30_-biotin-3′ was synthesized by incubating 1.0 mg of a reduced oligonucleotide composed of 30 adenines with a 5′-thiol (hexamethylene linker) and a 3′-biotin (triethylene glycol spacer) with 20 mM 2,2′-dithiodipyridine (Sigma) in acetonitrile for 2 h at room temperature (Supplementary Fig. 3b). The modified oligonucleotide was purified by ion-exchange chromatography on the monoQ FF Sepharose column.

### Thioredoxin purification^30^

The thioredoxin (Trx) mutants S1C-V5-C109 (S1C-A22P-I23V-C32S-C35S-P68A-C109) and S1C-V2-C109 (S1C-C32S-C35S-C109) were each cloned into the pET 30a+ plasmid (TopGene). *E. coli* BL21(DE3) cells (Novagen) were transformed with this plasmid and grown on LB medium supplemented with kanamycin (10 mg L^−1^) at 37 °C with shaking. During the exponential growth phase (OD_600nm_ = 0.6), protein production was induced with isopropyl-β-D-1-thiogalactopyranoside (0.4 mM, final concentration). After overnight growth, the cells were collected by centrifugation, suspended in TE buffer (30 mM Tris·HCl, 1 mM EDTA, pH 8.3) and lysed by sonication. Cell debris was removed by centrifugation, and the supernatant containing the protein was mixed with one quarter of its volume of 10% streptomycin sulfate (w/v) in TE buffer added drop by drop at 4 °C with stirring. After 2 h, the solution was centrifuged at 20,000 × *g* for 30 min, and the supernatant was loaded onto a Superdex 75 10/300 column (GE Healthcare). The peak corresponding to Trx was almost pure, but was further purified by ion-exchange chromatography on a monoQ FF column eluted with 0–1 M KCl in TE buffer. The purity of the protein was estimated with SDS-PAGE in 18% Criterion TGX Precast Midi Protein Gels (Bio-Rad) stained with InstantBlue Protein Stain (Expedeon). Protein concentration was estimated spectrophotometrically from the absorbance at 280 nm by using a molar extinction coefficient of 14,100 M^−1^cm^−1^ ^30^.

### Protein-oligonucleotide conjugates

The N-terminal cysteines of Trx as purified are not reactive because they are modified with aldehydes such as pyruvate^33^. The cysteines were deprotected by incubation with methoxyamine (0.4 M methoxyamine, 100 mM sodium phosphate, 150 mM NaCl, 5 mM TCEP, pH 7.0) at room temperature overnight. The protein was recovered with buffer exchange on a PD MiniTrap G-25 column (GE Healthcare). To modify the N-terminal cysteine with 5′-CBT-oligo(dC)_30_-biotin-3′, protein in 10-fold excess was mixed and incubated with 0.1–0.2 mg of the oligonucleotide in TE buffer (10 mM Tris·HCl, 1 mM EDTA, pH 7.5) at room temperature overnight. The 3′-biotin-oligo(dC)_30_-S1C-V5-C109 conjugate was purified by ion-exchange chromatography on a MonoQ FF Sepharose column eluted with 0–1 M KCl in the same buffer. The eluted conjugate was immediately mixed with 5′-S-thiopyridyl-oligo(dA)_30_-biotin-3′ and incubated for 16 h at room temperature^22^. The product (3′-biotin-oligo(dC)_30_-S1C-V5-C109-oligo(dA)_30_-biotin-3′) was purified by ion-exchange chromatography, and the mass was verified with SDS-PAGE and native MS (Supplementary Fig. 3).

### Purification and formation of heptameric α-hemolysin pores

α-Hemolysin (αHL) monomers were synthesized by in vitro transcription/translation and oligomerized into heptameric pores on rabbit red blood cell membranes^27^. [^35^S]Methionine radioactivity was used to identify the oligomeric band upon SDS-PAGE. The heptameric pores were eluted from the gel in a concentration range of 0.1–1 ng μL^−1^, ready to use in single-molecule experiments.

### Streptavidin variants expression and purification

Monovalent streptavidin (mSA) is a tetramer (SAe1D3) containing one streptavidin (SA) subunit with wild-type biotin binding affinity with a C-terminal Glu_6_ tag (SAe) and three “dead” subunits (D) with negligible biotin binding affinity^29,55^. Subunits were expressed as inclusion bodies in *E. coli*, solubilized in guanidinium hydrochloride, refolded by dilution, and the desired heterotetramer was purified by ion-exchange chromatography as described^55^. mSA assembly and composition were validated by mobility on SDS-PAGE with Coomassie Blue (Bio-Rad) staining for samples with or without boiling^55^. Monovalent traptavidin (mTA) is a tetramer (Tre1D3) containing one traptavidin (TA) subunit with a C-terminal Glu_6_ tag (Tre) and three “dead” subunits (D)^37,56^. mTA was purified and validated by using the same procedure as for mSA. Tetravalent SA had the subunit composition SAe4. Tetravalent TA had the subunit composition Tre4. Tetravalent forms were expressed and refolded as above, with purification by iminobiotin-Sepharose as described^55^.

### Single-molecule measurements^27^

A bilayer of 1,2-diphytanoyl-*sn*-glycero-3-phosphocholine (Avanti Polar Lipids) was made by using the Müller-Montal method on a 100 μm-diameter aperture made in a Teflon film (25 μm thick, Goodfellow) that separated two compartments (*cis* and *trans*). Each compartment was filled with electrolyte solution (10 mM HEPES, 2 M KCl, pH 7.2) and connected to the headstage of an Axopatch 200B amplifier (Molecular Devices) with Ag/AgCl electrodes. A lid was used to cover the compartments to avoid evaporation, and the temperature was controlled with a circulating bath to 23 °C. The signal from the amplifier was stored on a computer by using a Digidata 1440 A digitizer (Molecular Devices). Following membrane formation, heptameric αHL pores (0.2 μL, see above) were added to the *cis* compartment under an applied potential of +100 mV. After a single insertion event (identified by a step increase in the conductance of 1 nS) the *cis* compartment was perfused with fresh buffer by using a push-pull syringe driver PHD 2000 Syringe Pump (Harvard Apparatus) to prevent further insertions. Data were low-pass filtered at 5 kHz, which corresponds to a temporal resolution of ~0.2 ms, and sampled at 20 kHz. Voltage protocols in Clampex (Molecular Devices) were used to automatically change the voltage at defined times. The negative potential at level 7′ was maintained for 0.1 s, 0.5 s, 1 s, 1.5 s, 10 s, 100 s, or 1000 s, and therefore the protein was allowed to refold for 0.09 s, 0.49 s, 0.99 s, 1.49 s, 10 s, 100 s, or 1000 s, respectively (the average duration of the retro-translocation, level 6′, was deducted to calculate the refolding times at short timescales).

### Statistics and reproducibility

The state of individual molecules was probed hundreds of times under each condition. These data were collected on at least 3 independent experiments for each condition. Raw data files were first analyzed with pClamp (Molecular Devices) to obtain the current amplitude and dwell time in each level. The event histograms of the natural logarithm of the dwell times were fit to an exponential distribution by Igor Pro (Wavemetrics):

$$f\left( x \right) = Ake^{\left( {x - ke^x} \right)}$$

with: *A*, amplitude; *k*, rate constant; *x*, the natural logarithm of the dwell time. The rate constants in the main text are the best-fit values ± 1σ confidence intervals.

The 95% confidence interval in the estimation of the refolded fraction, *p*, (Fig. 3c) was estimated by using:

$$p \pm 1.96\sqrt {\frac{{p\left( {1 - p} \right)}}{n}}$$

with: *n*, the number of observations.

*k*-means clustering analysis was carried out by Igor Pro considering only the cases where both levels 2′ and 3′ were detected. In *k*-means clustering, the centroids define the center of each population (Supplementary Figs. 9, 11). Each data point is assigned to the population with the closest centroid. For Trx V5, a ~5% overestimation of the unfolded population and population A was assumed, due to an overlap with the population B. Therefore, the estimation of each population should be taken as a rough estimate. The different kinetic models used to explain the time-course evolution of refolding were evaluated with the application SimBiology from Matlab (MathWorks), which provided the Akaike Information Criterion (AIC) and the Bayesian Information Criterion (BIC) (Supplementary Figs. 10, 12).

### Reporting summary

Further information on research design is available in the Nature Research Reporting Summary linked to this article.

## Supplementary information

Supplementary Information

Reporting Summary

## Data Availability

Relevant data and/or materials are available upon reasonable request from D.R-L. and/or H.B.
