# Supplementary material for: Transmembrane protein rotaxanes reveal kinetic traps in the refolding of translocated substrates
Source: Commun Biol. 2020 Apr 3;3:159. doi: 10.1038/s42003-020-0840-5 (PMC7125113; doi:10.1038/s42003-020-0840-5)
Supplement: Supplementary file 1 — Supplementary Information [file 42003_2020_840_MOESM1_ESM.pdf]

Supplementary Figures

Supplementary Figure 1

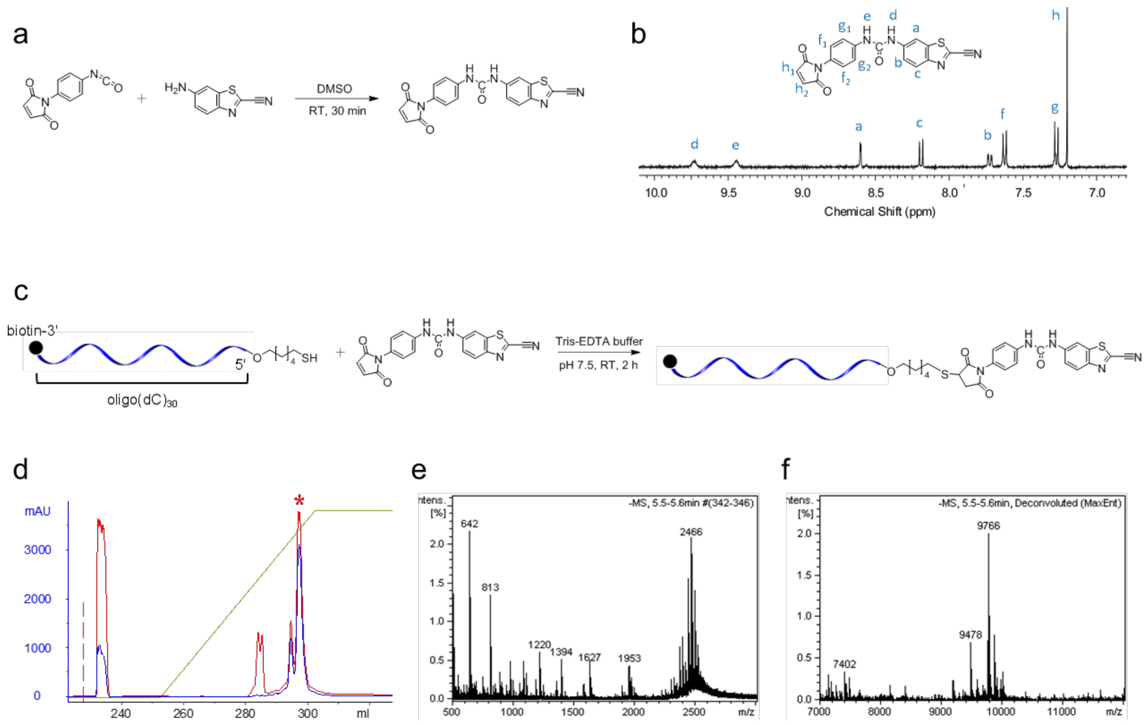

Supplementary Figure 1. Synthesis of 5'-CBT-oligo(dC)$_{30}$-biotin-3'. a Reaction between 6-amino-2-CBT and *p*-maleimidophenyl isocyanate to form the maleimide-CBT crosslinker. **b** ^{1}H NMR spectrum of the product (400 MHz, DMSO-d$_6$): δ 7.20 (s, 2H), 7.26-7.28 (d, 2H, J = 8.85), 7.61-7.63 (d, 2H, J = 8.85), 7.72-7.74 (dd, 1H, J = 9.00, 1.98), 8.18-8.20 (d, 1H, J = 9.00), 8.60-8.61 (d, 1H, J = 1.98), 9.44(s, 1H), 9.73(s, 1H). **c** Reaction of 5'-thiol-oligo(dC)$_{30}$-biotin-3' with the maleimide-CBT crosslinker. **d** Ion-exchange chromatography of the modified oligonucleotide (absorbance at 280 nm, blue; 254 nm, red). **e** ESI-MS of the major peak (*) from 'd'. **f** Deconvoluted ESI-MS: calculated for 5'-CBT-oligo(dC)$_{30}$-biotin-3' = 9,769; observed = 9,766.

Supplementary Figure 2

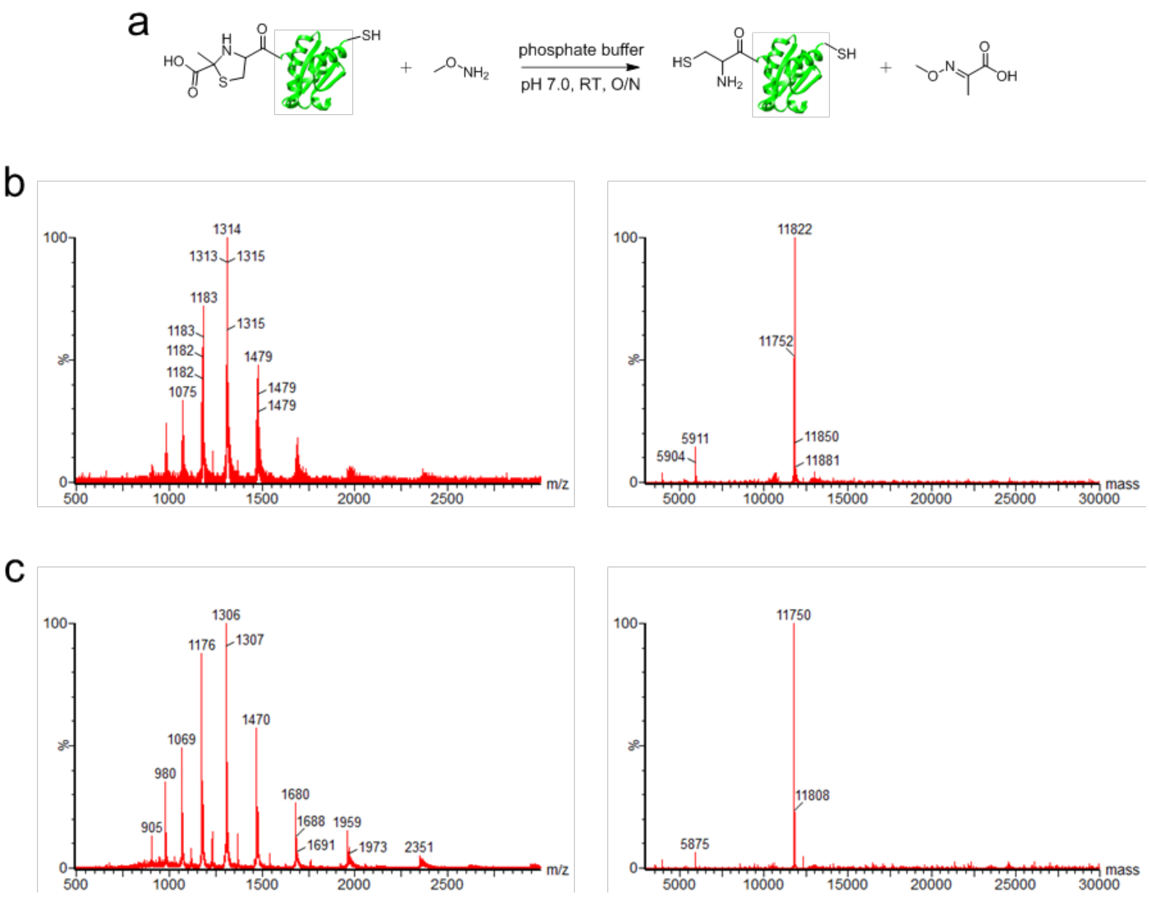

Supplementary Figure 2. Removal of pyruvate from the N-terminal cysteine of Trx S1C-V5-C109. a Pyruvate was removed from the N terminus of Trx by incubation with methoxyamine. **b** ESI-MS, and the deconvoluted spectrum, of Trx as purified from *E. coli*. Calculated $[M + H]^+$ = 11,749. The observed mass of 11,822 corresponds to the pyruvate adduct (+73 Da). **c** ESI-MS, and the deconvoluted spectrum, after the incubation with methoxyamine. Observed = 11,750.

Supplementary Figure 3

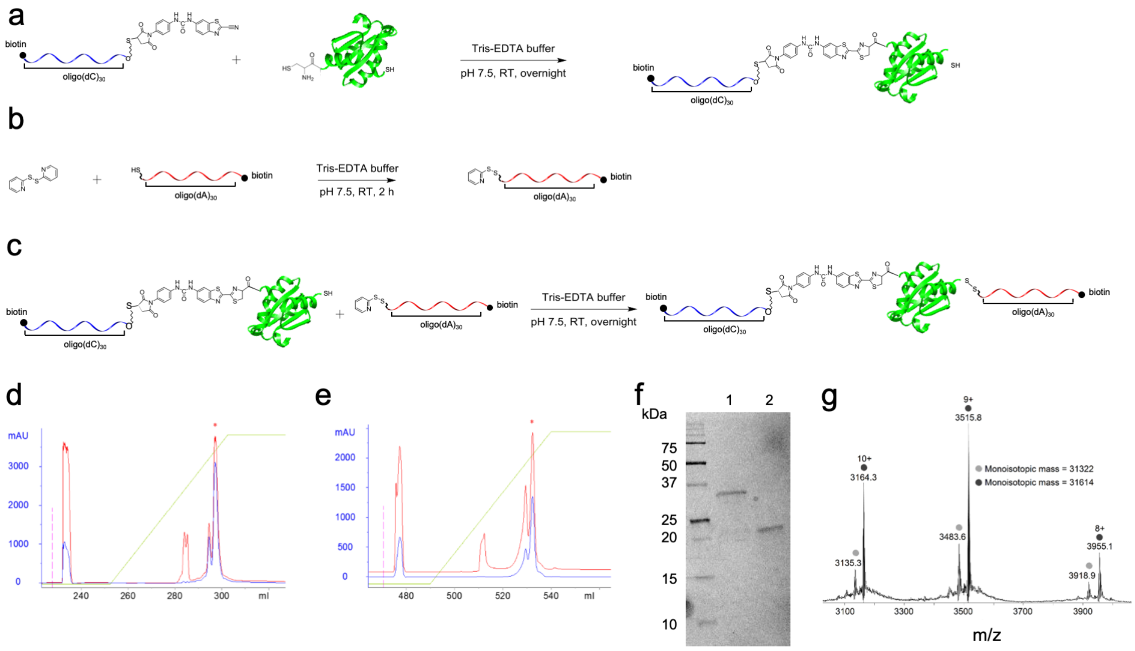

Supplementary Figure 3. Synthesis of 3'-biotin-oligo(dC)$_{30}$-S1C-V5-C109-oligo(dA)$_{30}$-biotin-3'. a The 30-mer cytosine oligonucleotide (5'-CBT-oligo(dC)$_{30}$-biotin-3') was coupled to the N-terminal cysteine of Trx S1C-V5-C109. **b** A 30-mer adenine oligonucleotide (5'-thiol-oligo(dA)$_{30}$-biotin-3') was activated with 2,2'-dipyridyl disulphide. **c** The 3'-biotin-oligo(dC)$_{30}$-S1C-V5-C109 conjugate produced in 'a' was reacted with the activated oligonucleotide from 'b' to form the 3'-biotin-oligo(dC)$_{30}$-S1C-V5-C109-oligo(dA)$_{30}$-biotin-3' conjugate. **d** Ion-exchange chromatography of the reaction products shown in 'a'. The asterisk marks the elution of the 3'-biotin-oligo(dC)$_{30}$-S1C-V5-C109 conjugate (absorbance at 280 nm, blue; 254 nm, red). **e** Ion-exchange chromatography of the reaction products shown in 'c'. The asterisk marks the elution of the 3'-biotin-oligo(dC)$_{30}$-S1C-V5-C109-oligo(dA)$_{30}$-biotin-3' conjugate. **f** SDS-PAGE of the purified products. Lane 1: the 3'-biotin-oligo(dC)$_{30}$-S1C-V5-C109-oligo(dA)$_{30}$-biotin-3' conjugate; lane 2: the 3'-biotin-oligo(dC)$_{30}$-S1C-V5-C109 conjugate. **g** Native MS of the 3'-biotin-oligo(dC)$_{30}$-S1C-V5-C109-oligo(dA)$_{30}$-biotin-3' conjugate. Calculated = 31,598; observed = 31,614. The difference observed is due to the opening of the succinimide ring by hydrolysis (+18). The minor peak of 31,322 corresponds to a conjugate missing one cytosine nucleotide.

Supplementary Figure 4

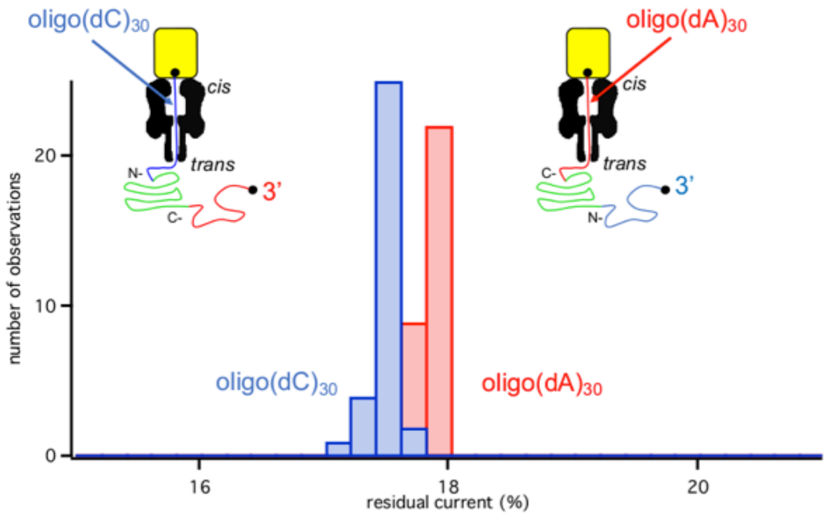

Supplementary Figure 4. The residual current in level 5 depends on the threading direction. Histogram of the residual currents obtained from oligo(dC)$_{30}$-V5-oligo(dA)$_{30}$ that threaded N terminus-first (blue bars) or C terminus-first (red bars) in the presence of equimolar amounts of mSA. After translocation of the protein through the pore, oligo(dC)$_{30}$ is arrested within the pore by mSA if the translocation is N terminus-first. Oligo(dA)$_{30}$ is arrested within the pore if the translocation is C terminus-first. The different base compositions produce different residual currents. Data were collected with two different αHL pores. The residual currents were $I_{res\%}$ = 17.8 $\pm$ 0.1 for oligo(dA)$_{30}$ and $I_{res\%}$ = 17.4 $\pm$ 0.1 for oligo(dC)$_{30}$ (mean $\pm$ standard deviation; best-fit values to Gaussian distributions).

Supplementary Figure 5

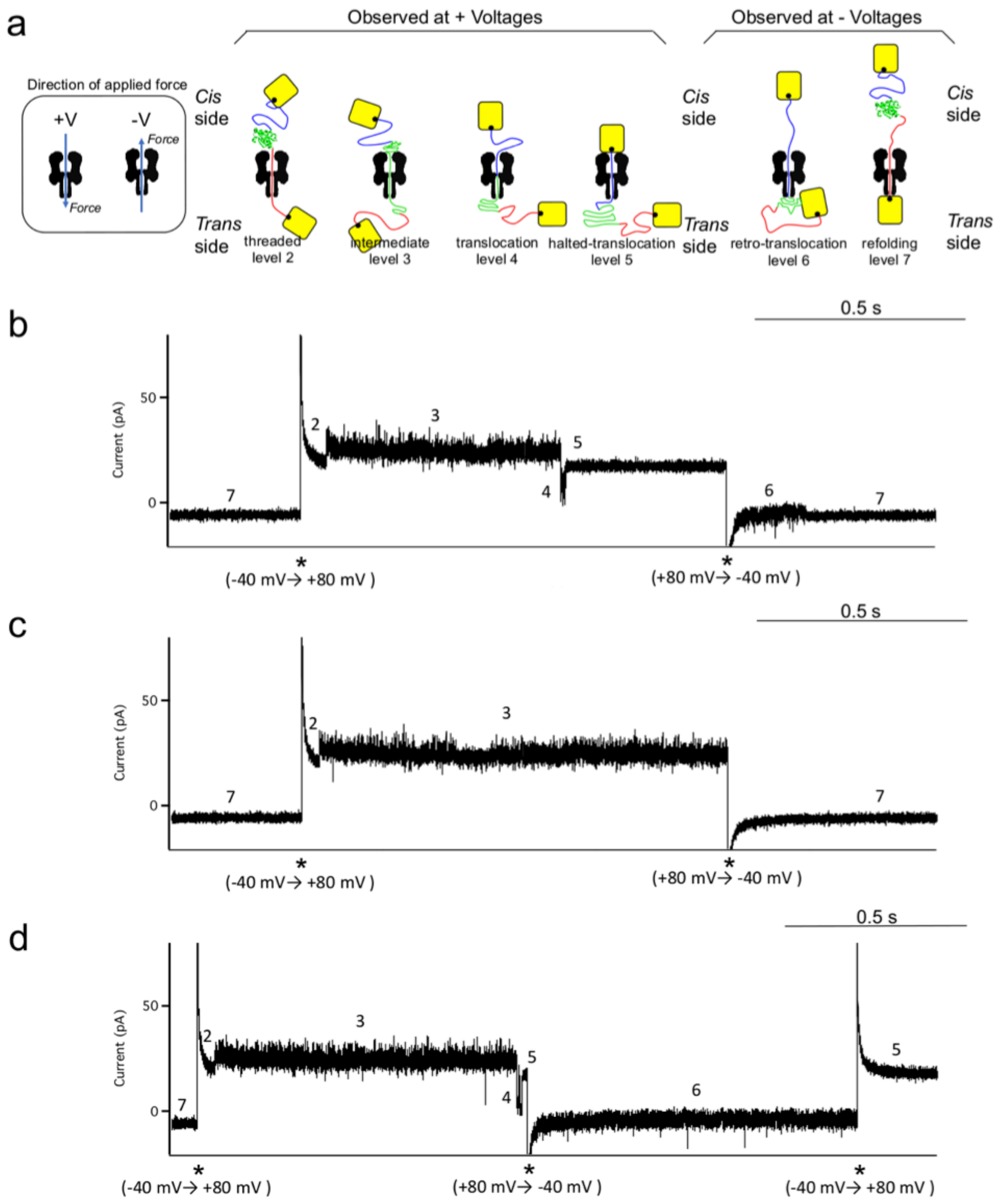

Supplementary Figure 5. Ionic current signals during movement of the oligo(dC)$_{30}$-V5-oligo(dA)$_{30}$ within the αHL pore. a Current levels associated with different states of the rotaxane. Box: change in the direction of the force acting on an oligonucleotide arising from the reversal of voltage polarity. **b** Ionic current signal showing one cycle of unfolding-translocation-refolding. The asterisk indicates a change in the voltage polarity. Initially, under a negative voltage, the protein is located in the *cis* compartment (level 7'). A change in the voltage polarity to +80 mV pulls the protein through the pore towards the *trans* compartment. The protein unfolds (levels 2' and 3') and translocates (level 4'), and oligo(dC)$_{30}$-V5-oligo(dA)$_{30}$ is then arrested by mSA with the protein in the *trans* compartment (level 5'). A switch in the voltage polarity pulls the protein

back towards the *cis* compartment. The protein, if refolded, unfolds and retro-translocates from the *trans* to the *cis* compartment (level 6'), and during level 7', it refolds in the *cis* compartment before a new cycle is initiated. **c** If the voltage is switched from positive to negative during level 3' (in which the protein has not reached the *trans* compartment) only level 7' (but not level 6') is observed. **d** If the voltage is switched from negative to positive during level 6', level 5' follows, because the protein has not completed retro-translocation into the *cis* compartment.

Supplementary Figure 6

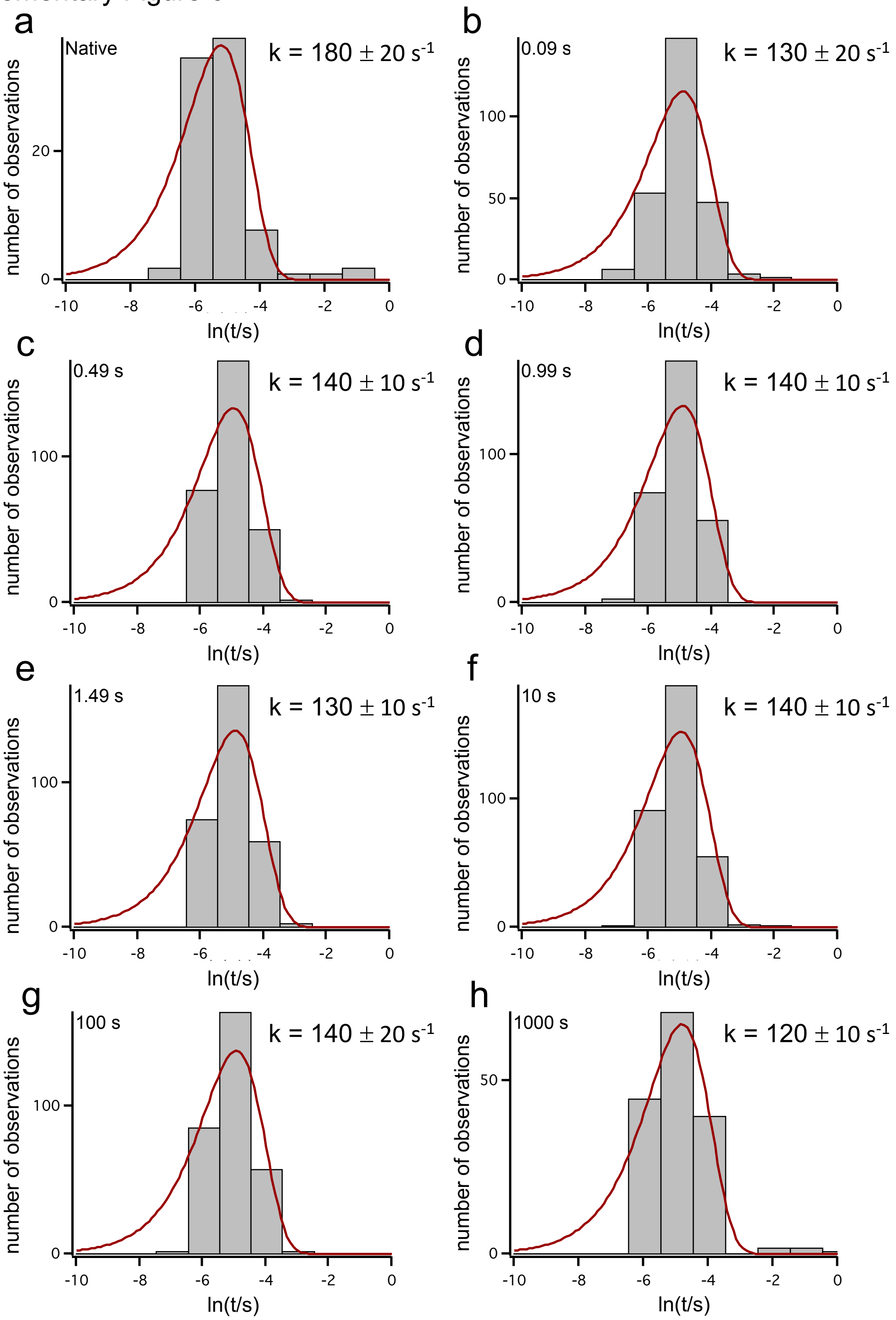

Supplementary Figure 6. The translocation time of the unfolded protein (dwell time in level 4') does not depend on the refolding time (dwell time in level 7'). a Histogram of the dwell time in level 4 during co-translocational unfolding of native Trx V5 at +100 mV. The red line is the best fit to an exponential distribution. **b** Histogram of the dwell time in level 4' during co-translocational unfolding of refolded Trx V5 after a refolding time of 0.09 s. The red line is the best fit to an exponential distribution. **c** Same as 'b' for a refolding time of 0.49 s. **d** A refolding time of 0.99 s. **e** A refolding time of 1.49 s. **f** A

refolding time of 10 s. **g** A refolding time of 100 s. **h** A refolding time 1000 s. Rate constants ($k_{4\rightarrow1}$ or $k_{4'\rightarrow5'}$) are indicated within each panel (best-fit value ± 1σ confidence interval). Histograms are based on data collected from at least three independent experiments.

Supplementary Figure 7

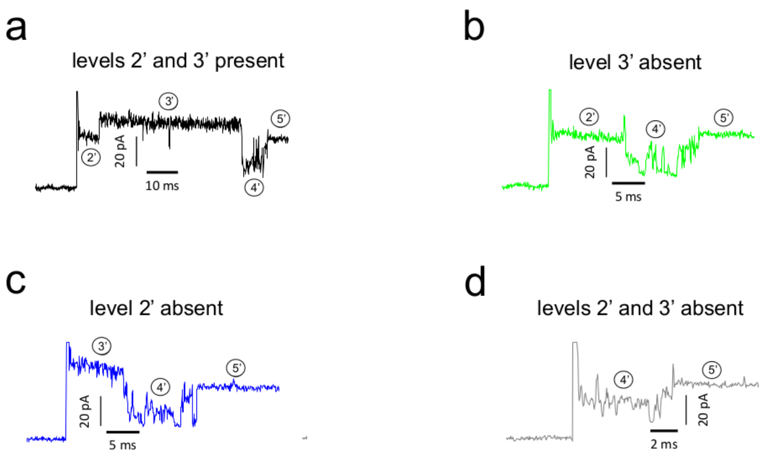

Supplementary Figure 7. Presence and absence of levels 2' and 3' upon co-translocational unfolding of the refolded state. Representative traces of ionic currents obtained from the oligo(dC)$_{30}$-V5-oligo(dA)$_{30}$/αHL rotaxane at +100 mV after 1.49 s of refolding during level 7' at -100 mV. **a** both levels 2' and 3' are present. **b** level 3' is absent, which suggests either that the N-terminal domain of the protein did not refold or that it unfolded faster than the detection limit (0.2 ms, a short spike is observed at the end of level 2', which suggests that the second explanation may apply here). **c** level 2' is not observed, which suggests either that the C-terminal domain did not refold or that it unfolded faster than the detection limit (here the capacitive transient after the voltage step increases the detection limit to ~1 ms). **d** neither level 2' nor 3' is observed, which suggests either that the protein remained unfolded or that it unfolded faster than the detection limits.

Supplementary Figure 8

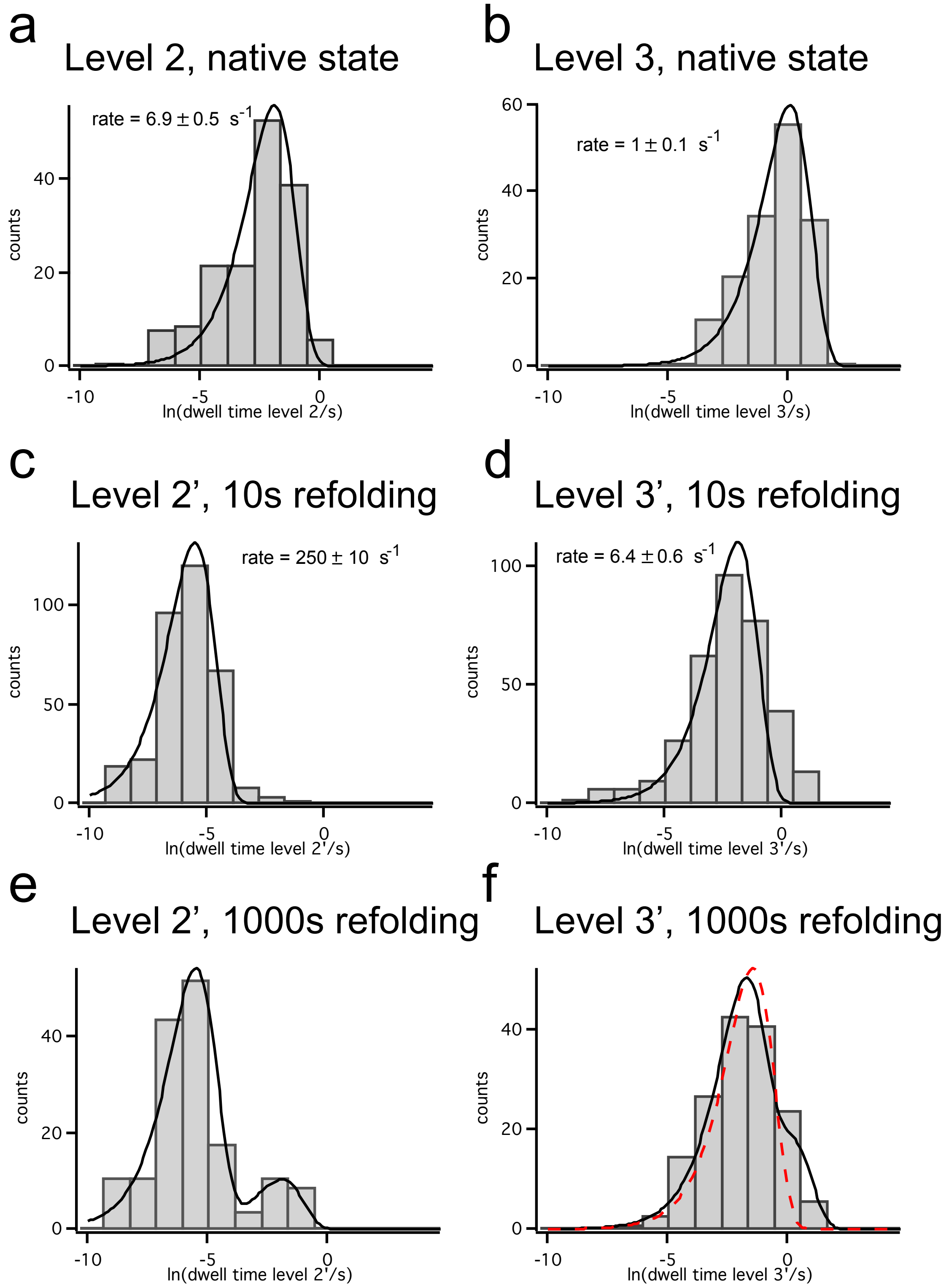

Supplementary Figure 8. Dwell time distributions in levels 2' and 3'. Histograms showing the dwell time distributions in levels 2' and 3' for the oligo(dC)$_{30}$-V5-oligo(dA)$_{30}$/αHL rotaxane at +100 mV. **a** Dwell time in level 2 for the native state. The solid line is the best fit to an exponential distribution. **b** Dwell time in level 3 for the native state. **c** Dwell time in level 2' after 10 s of

refolding during level 7'. **d** Dwell time in level 3' after 10 s of refolding during level 7'. **e** Dwell time in level 2' after 1000 s of refolding during level 7'. The solid line is the best fit to a double exponential distribution using the rate constants obtained from 'a' and 'c'. **f** Dwell time in level 3' after 1000 s of refolding during level 7'. The solid line is the best fit to a double exponential distribution using the rate constants obtained from 'b' and 'd'. The red dashed line is a fit to a single exponential distribution. The derived rate constants are indicated within each panel (best-fit value ± 1σ confidence interval). Histograms are based on data collected from at least three independent experiments.

Supplementary Figure 9

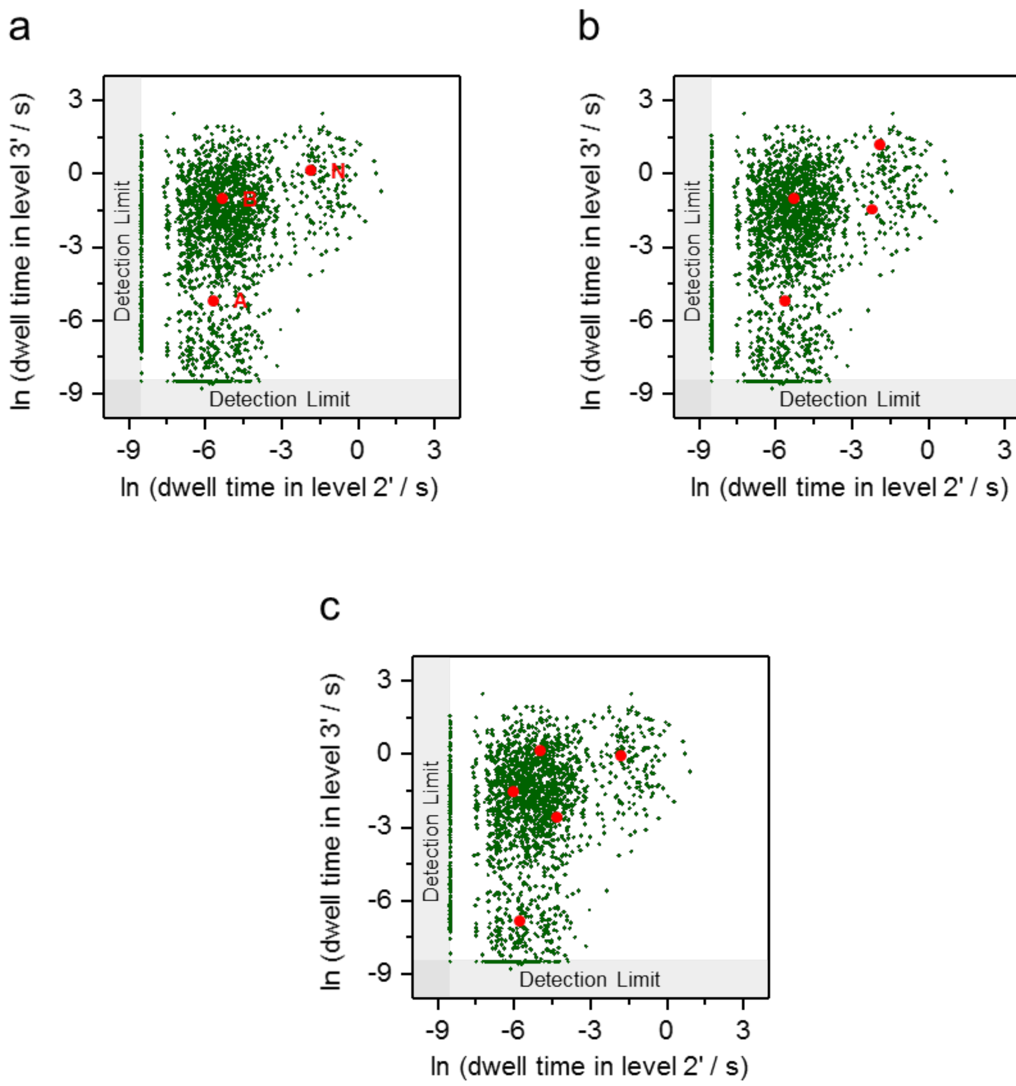

Supplementary Figure 9. Centroids obtained by *k*-means clustering of dwell times with the oligo(dC)$_{30}$-V5-oligo(dA)$_{30}$/αHL rotaxane. The natural logarithm of the dwell times in levels 2' and 3' for the various refolding times combined, together with those in levels 2 and 3 for the native protein, are used to obtain the centroids of three different populations. Each data point (green) is assigned to the population with the closest centroid (red). **a** Centroids obtained with *k*-means clustering when three clusters are considered. The lowest centroid is shifted towards the upper cluster because the upper cluster comprises more data points. **b** Considering four clusters does not improve the assignment to the lowest population. **c** Considering five clusters gives centroids that clearly identify the different populations, with the major population having three centroids. Data were obtained from three different pores for the native protein and three for the refolded protein.

Supplementary Figure 10

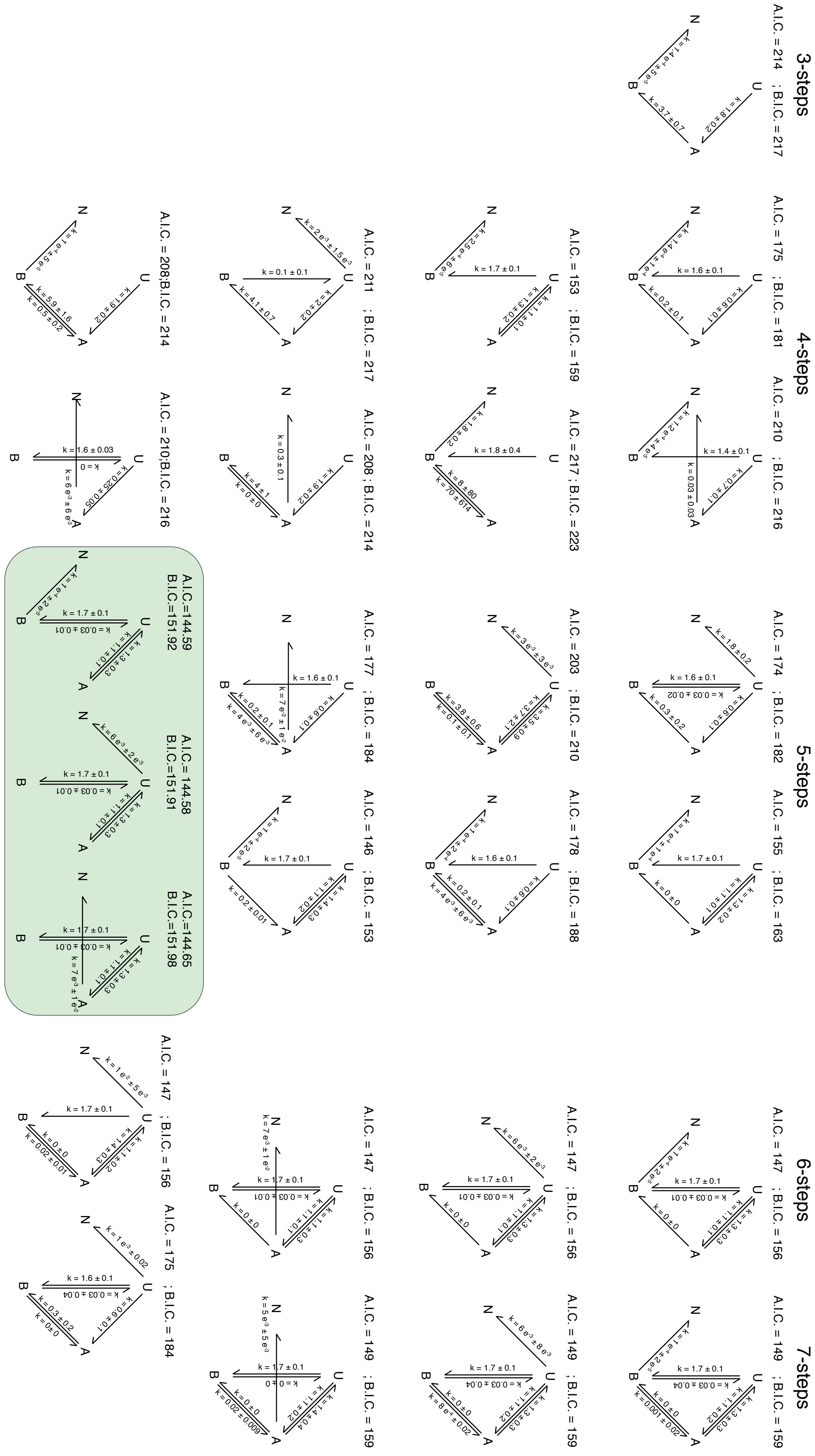

Supplementary Figure 10. The best models describing the folding kinetics of Trx V5 have five kinetic steps and two kinetic traps. The models considered assume that formation of the native state is irreversible and that, at infinite time, all molecules reach the native state (i.e. there is no irreversible formation of the populations A and B). The models displayed fit the time-dependent evolution of populations A and B, the native state and the unfolded state. For each model, the BIC and the AIC are indicated. For both criteria, the lowest value indicates the model that better fits the data with fewer parameters. Three models that consider the populations A and B to be in equilibrium with the unfolded state have the lowest BIC and AIC values (green box). The lowest one -therefore the chosen one-, the formation of the native state only occurs from the unfolded state, and both populations A and B are off-pathway.

Supplementary Figure 11

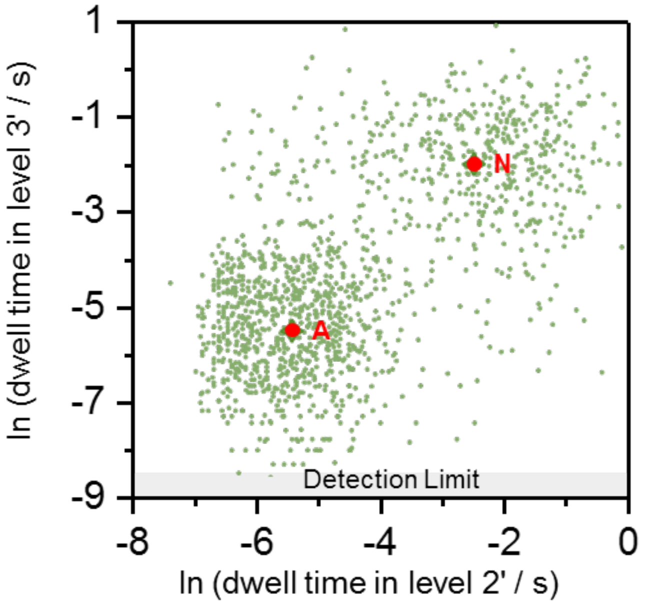

Supplementary Figure 11. Centroids obtained by *k*-means clustering on dwell times with the oligo(dC)$_{30}$-V2-oligo(dA)$_{30}$/αHL rotaxane. The natural logarithm of the dwell times in levels 2' and 3' for the various refolding times combined, together with those in level 2 and 3 for the native state, are used to obtain the centroids (red) of two different populations. Data were obtained from three different pores for the native protein and three for the refolded protein.

Supplementary Figure 12

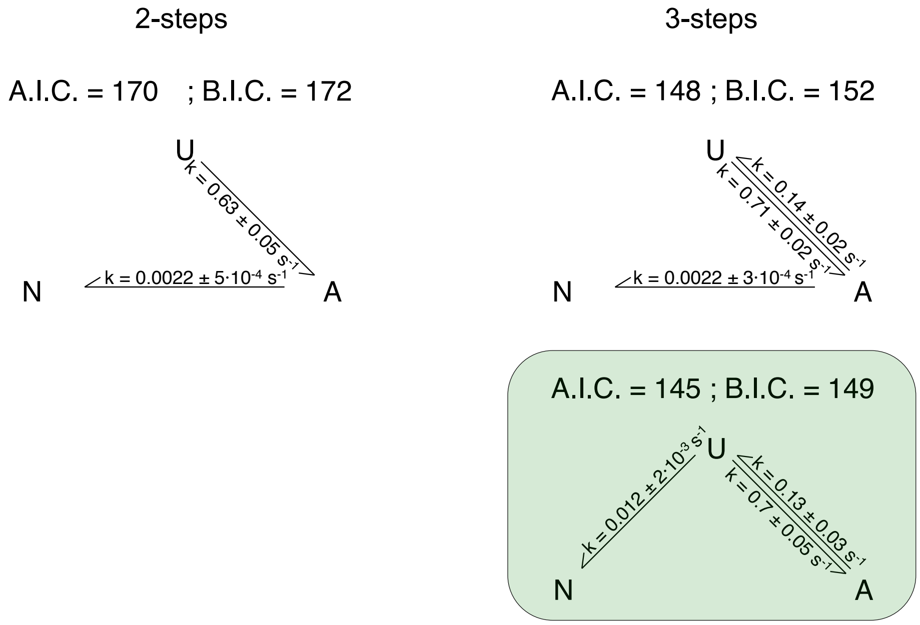

Supplementary Figure 12. The best model describing the folding kinetics of Trx V2 has three kinetic steps and one kinetic trap. The models considered assume that formation of the native state is irreversible and that, at infinite time, all molecules reach the native state (i.e. there is no irreversible formation of the population A). The presented models were used to fit the time-dependent evolution of population A, the native state and the unfolded state. For each model, the BIC and the AIC are shown. For both criteria, the lowest value indicates the model that better fits the data with fewer parameters. The best-fit model is in the box and indicates that the native state only occurs from the unfolded state and the population A is off-pathway.
